# Identification of key genes for HNSCC from public databases using bioinformatics analysis

**DOI:** 10.1186/s12935-021-02254-7

**Published:** 2021-10-18

**Authors:** Yuchu Ye, Jingyi Wang, Faya Liang, Pan Song, Xiaoqing Yan, Sangqing Wu, Xiaoming Huang, Ping Han

**Affiliations:** 1 Sun Yat-Sen Memorial Hospital, Department of Otolaryngology Head and Neck Surgery, Sun Yat-Sen University, Guangzhou, China; 2 Guangdong Provincial Key Laboratory of Malignant Tumor Epigenetics and Gene Regulation, Sun Yat-Sen Memorial Hospital, Sun Yat-Sen University, Guangzhou, China

**Keywords:** Head and neck squamous cell carcinoma (HNSCC), GEO database, The Cancer Genome Atlas (TCGA), Integrated bioinformatics, DEG (differentially expressed gene) analysis

## Abstract

**Background:**

The cause and underlying molecular mechanisms of head and neck squamous cell carcinoma (HNSCC) are unclear. Our study aims to identify the key genes associated with HNSCC and reveal potential biomarkers.

**Methods:**

In this study, the expression profile dataset GSE83519 of the Gene Expression Omnibus database and the RNA sequencing dataset of HNSCC of The Cancer Genome Atlas were included for analysis. Sixteen differentially expressed genes were screened from these two datasets using R software. Gene Expression Profiling Interactive Analysis 2 (GEPIA2) was then adopted for survival analysis, and finally, three key genes related to the overall survival of HNSCC patients were identified. Furthermore, we verified these three genes using the Oncomine database and from real-time PCR and immunohistochemistry results from HNSCC tissues.

**Results:**

The expression data of 44 samples from GSE83519 and 545 samples from TCGA-HNSC were collected. Using bioinformatics, the two databases were integrated, and 16 DEGs were screened out. Gene Ontology (GO) enrichment analysis showed that the biological functions of DEGs focused primarily on the apical plasma membrane and regulation of anoikis. Kyoto Encyclopedia of Genes and Genomes (KEGG) signalling pathway analysis showed that these DEGs were mainly involved in drug metabolism-cytochrome P450 and serotonergic synapses. Survival analysis identified three key genes, CEACAM5, CEACAM6 and CLCA4, that were closely related to HNSCC prognosis. The Oncomine database, qRT–PCR and IHC verified that all 3 key genes were downregulated in most HNSCC tissues compared to adjacent normal tissues.

**Conclusions:**

This study indicates that integrated bioinformatics analyses play an important role in screening for differentially expressed genes and pathways in HNSCC, helping us better understand the biomarkers and molecular mechanism of HNSCC.

## Background

Head and neck cancers, with over 800,000 new cases each year, are among the most common malignancies in the world [1]. Squamous cell carcinoma accounts for more than 90% of these cancers. Continuous exposure to tobacco, tobacco-like products and alcohol is thought to increase the risk of head and neck squamous cell carcinoma (HNSCC) [2]. The crucial treatment strategy includes surgery, radiotherapy and chemotherapy. However, once diagnosed with distant metastasis, the median survival time is only 3.3–3.9 months, and the mortality rate of HNSCC remains high [3, 4]. The incidence of tumour recurrence after standard treatment is 15–50% [4]. In addition to HPV status, biomarkers for precise targets of HNSCC treatment have yet to be elucidated [5].

Bioinformatics, a combination of molecular biology and information technology, has become a crucial tool for understanding the molecular mechanisms and signalling pathways of cancers. The development of bioinformatics technology and the identification of biomarkers have enabled great progress in the diagnosis and treatment of cancers, such as HNSCC [6, 7]. Gene expression profiling technologies, including RNA sequencing (RNA-seq) [8] and microarray profiling, have been used to uncover molecular variations in cancers versus adjacent noncancerous tissues. Molecular-level data mining from different databases can help oncologists discover tumour markers for clinical diagnosis or therapy [9]. The vast majority and the most representative bioinformatics works can be obtained from two databases, Gene Expression Omnibus (GEO) and The Cancer Genome Atlas (TCGA).

Numerous studies [6, 10, 11] have demonstrated that the occurrence and development of HNSCC are closely associated with the mutation and abnormal expression of genes, which include Six genes (PEX11A, NLRP2, SERPINE1, UPK, CTTN, D2HGDH) signature, and mutations in 4 genes (KL, CCR7, LGR5, RORB) are associated with prognosis of HNSCC.

In this study, we analysed sequencing data from the GEO and TCGA databases to screen differentially expressed genes (DEGs) of HNSCC. Further exploration focused on the expression profiles of these DEGs in cancer tissues originating from the oropharynx, hypopharynx and larynx and corresponding adjacent noncancerous tissues. These results may help demonstrate the molecular mechanism and discover potential therapeutic targets of HNSCC.

## Methods

Microarrays are the main technique in the postgenomic era used to analyse global gene expression profiles. Specially designed arrays can also detect single-nucleotide polymorphisms or fusion genes and can be used to draw exon junction diagrams [12].

Compared with RNA-seq technology, the advantages of microarray technology include a more regular calculation method in which the gene expression levels are proportional to the degree of probe hybridization, as well as a lower length bias. [13]. Typically, real-time PCR or proteomic methods are used for validation of DEGs [13, 14]. Due to the rapid progress of bioinformatics science, by comparing the results from different databases and platforms, DEG validation can be completed conveniently. Microarray data analysis has become easier due to the development of various software packages.

### Microarray data

The Gene Expression Omnibus (http://www.ncbi.nlm.nih.gov/geo, GEO), one of the most famous public genome data repositories, consists of high-throughput gene expression data, microchips, and microarrays [15, 16]. Gene Platform (GPL) and Gene Series (GSE) comprise the GEO data. Using the keyword “head and neck squamous cell carcinoma” to search the GEO database, the gene expression dataset GSE83519 (not published, gene expression data can be obtained from https://www.ncbi.nlm.nih.gov/geo/query/acc.cgi?acc=GSE83519), which contains HNSCC samples and adjacent paired normal tissues from 22 patients, was selected from GEO. A GPL4133 Agilent-014850 Whole Human Genome Microarray 4 × 44 K G4112F (Feature Number version) was used for the GSE83519 platform. The platform and series matrix data were downloaded as TXT files.

At the same time, all 544 sets of RNA-seq data from 500 patients of the HNSCC project of The Cancer Genome Atlas (TCGA-HNSC) and their clinical information, including 44 paired tumour and adjacent noncancerous tissues, were downloaded [17] to calculate the mRNA expression. Our results were completely based on TCGA Research Network: https://www.cancer.gov/tcga.

Statistical background correction, normality standardization and expression level calculation were performed to make the downloaded data comparable using R software (× 64 3.6.1) and the Limma package.

### Identification and function enrichment analysis of DEGs

R software and the packages Impute and Limma were used together to calculate the expression values of the genes in GSE83519 and TCGA-HNSC, respectively. The log fold-change (logFC) values between HNSCC tissues and adjacent noncancerous tissues were calculated. DEGs were considered to be significant when their logFC ≥ 1 or ≤ − 1 and adjusted P value < 0.05. Heat and volcanic maps of DEGs from the two databases were constructed with several R software packages, including Pheatmap, ggplot, and ggplot2. Subsequently, the intersecting DEGs (IDEGs) from the two datasets above were screened for detailed analysis using the Venn Diagram package.

Gene Ontology (GO) provides an overall framework to describe the functions of genes from different organisms [18]. GO annotation includes three categories: biological process (BP), cellular component (CC) and molecular function (MF). The genes added to the analysis will be assigned to one of the above three categories according to their functions in the cell.

Kyoto Encyclopedia of Genes and Genomes (KEGG) consists of fifteen manually differentiated datasets. The KEGG PATHWAY dataset is the main dataset in the KEGG project [19]. KEGG PATHWAY assigns gene sets from molecular-level functions to higher-level functions through different pathways and can be used for both functional explanation or forecasting of genes of interest and practical applications of genomic information.

To reveal further biological significance behind the IDEGs screened out in our study, we used the packages DOSE, GO.db, topGO, and clusterProfiler and other R packages to perform GO function as well as the KEGG pathway enrichment analyses in the IDEGs collected from the Venn Diagram package. An adjusted *P*-value and *q* value < 0.05 were considered statistically significant.

### PPI and survival analyses of DEGs

To further reveal the molecular mechanism of HNSCC at the protein level, protein–protein interaction (PPI) analysis of IDEG production was performed using String11.0 (http://string-db.org), a database consisting of known and forecasted PPIs of humans and other species [20].

To further evaluate and reveal the relationship between the DEG expression level and HNSCC prognosis, GEPIA2 was used for IDEG survival analysis. An updated version of GEPIA was used to analyse the correlation between gene expression and survival data from the TCGA and GTEx projects [21]. This website also provides a variety of methods, such as differential expression gene analysis between tumour and normal tissues, similar gene detection, and correlation expression analysis [21], for genetic-level data analyses. For the results of log-rank survival analysis, the criterion of statistically significant difference between two expression level groups was *P* < 0.05. IDEGs significantly associated with HNSCC prognosis were identified as the key genes in our study.

### Verification of key genes with oncomine

Another online software program, Oncomine (https://www.oncomine.org), was used to verify our key genes [22]. The following screening criteria were set: (1) “Gene: gene names of key genes”; (2) “Analysis Type: Cancer vs. Normal Analysis”; and (3) “Cancer Type: Head and Neck Cancer”. The criteria of the key genes were as follows: differential expression between head and neck cancers and normal tissues and similar expression trends to those of our original databases, fold change ≥ 2, and *P* < 0.05.

### Specimens

Between Dec 2018 and May 2019, 15 HNSCC patients were enrolled in this study at the authors’ institution. The clinicopathological features of the 15 patients are listed in Table 1. HNSCC tissues and paired adjacent noncancerous tissues located 20 mm away from the tumour margin were obtained, including five cases of laryngeal carcinoma (LC), five cases of hypopharyngeal carcinoma (HPC) and five cases of oropharyngeal carcinoma (OPC). Written informed consent was provided by all patients for the collection of surgical specimens. The study was approved by the Ethics Committee of the authors’ institution and was accomplished in accordance with the Declaration of Helsinki [23].

**Table 1:** Clinicopathological characteristics of the 15 patients

Type	No.	Age (years)	Sex	Tumour site	Stage
Laryngeal carcinoma	1	73	Male	Glottic	T2N0M0
	2	61	Male	Supraglottic	T3N0M0
	3	61	Male	Glottic	T4N2M0
	4	54	Male	Supraglottic	T4N2M0
	5	57	Male	Supraglottic	T1N2M0
Hypopharyngeal carcinoma	1	67	Male	Pyriform fossa	T4N2M0
	2	51	Male	Pyriform fossa	T4N2M0
	3	61	Male	Pyriform fossa	T4N2M0
	4	52	Male	Pyriform fossa	T3N0M0
	5	72	Male	Pyriform fossa	T3N0M0
Oropharyngeal carcinoma	1	59	Male	Right tonsil	T3N2M0
	2	54	Male	Right tonsil	T3N2M0
	3	39	Male	Right tonsil	T2N0M0
	4	67	Male	Tongue base	T3N3M0
	5	68	Male	Right tonsil	T4N2M0

### Real-time PCR

TRIzol reagent (Invitrogen, USA) was used to extract total RNA from the tissues. cDNA obtained from the reverse transcription of the RNA was used as templates for detecting the expression levels of the three key genes we identified, carcinoembryonic antigen-related cell adhesion molecule-5 (CEACAM5), carcinoembryonic antigen-related cell adhesion molecule-6 (CEACAM6) and chloride channel accessory 4 (CLCA4). A CFX96 Touch sequence detection system (Bio–Rad, USA) was used for real-time PCR with SYBR Green (Invitrogen) and subsequent data collection. Real-time PCR detection was normalized to β-actin (ACTB). All experiments were carried out in triplicate independently to eliminate system errors. Differences in relative expression levels were analysed through Student’s t-test using GraphPad Prism software. The results were considered statistically significant when p < 0.05.The primer sequences of CEACAM5, CEACAM6 and CLCA4 were as follows:

CEACAM5 Forward 5′-ATCCTATACGTGCCAAGCCC-3′.

Reverse 5′-ATGAAGGGTTTGGGTGGCTC-3′.

CEACAM6 Forward 5′-ACAGTCTCTGGAAGTGCTCC-3′.

Reverse 5′-TGGCCAGCACTCCAATCG-3′.

CLCA4 Forward 5′-AGGGGAGAAAAACAGCATGGAG-3′.

Reverse 5′-CCACATTCTGTGAACTGCTTGG-3′.

ACTB Forward 5′-TATGACAACAGCCTCAAGAT-3′.

Reverse 5′-AGTCCTTCCACGATACCA-3′.

### Immunohistochemistry (IHC)

IHC was performed for 15 pairs of clinically diagnosed HNSCC tissues and adjacent noncancerous tissues with rabbit anti-CEACAM6 antibody (1:1000; ab134074, Abcam, UK), mouse anti-CEACAM5 antibody (1:400; CST2383, Cell Signaling Technology, USA), and rabbit anti-CLCA4 antibody (1:60 dilution, ab197347, Abcam, UK) as primary antibodies. A biotinylated secondary antibody (anti-rabbit and anti-mouse IgG, Zymed Laboratories, USA) was used according to the manufacturer’s protocols as previously described [24]. The semiquantification analysis of IHC results was estimated with the criteria described in our previous publication [25]. All results were confirmed and verified by two pathology experts.

## Results

### Microarray data and identification of DEGs from two datasets

**Fig. 1:**
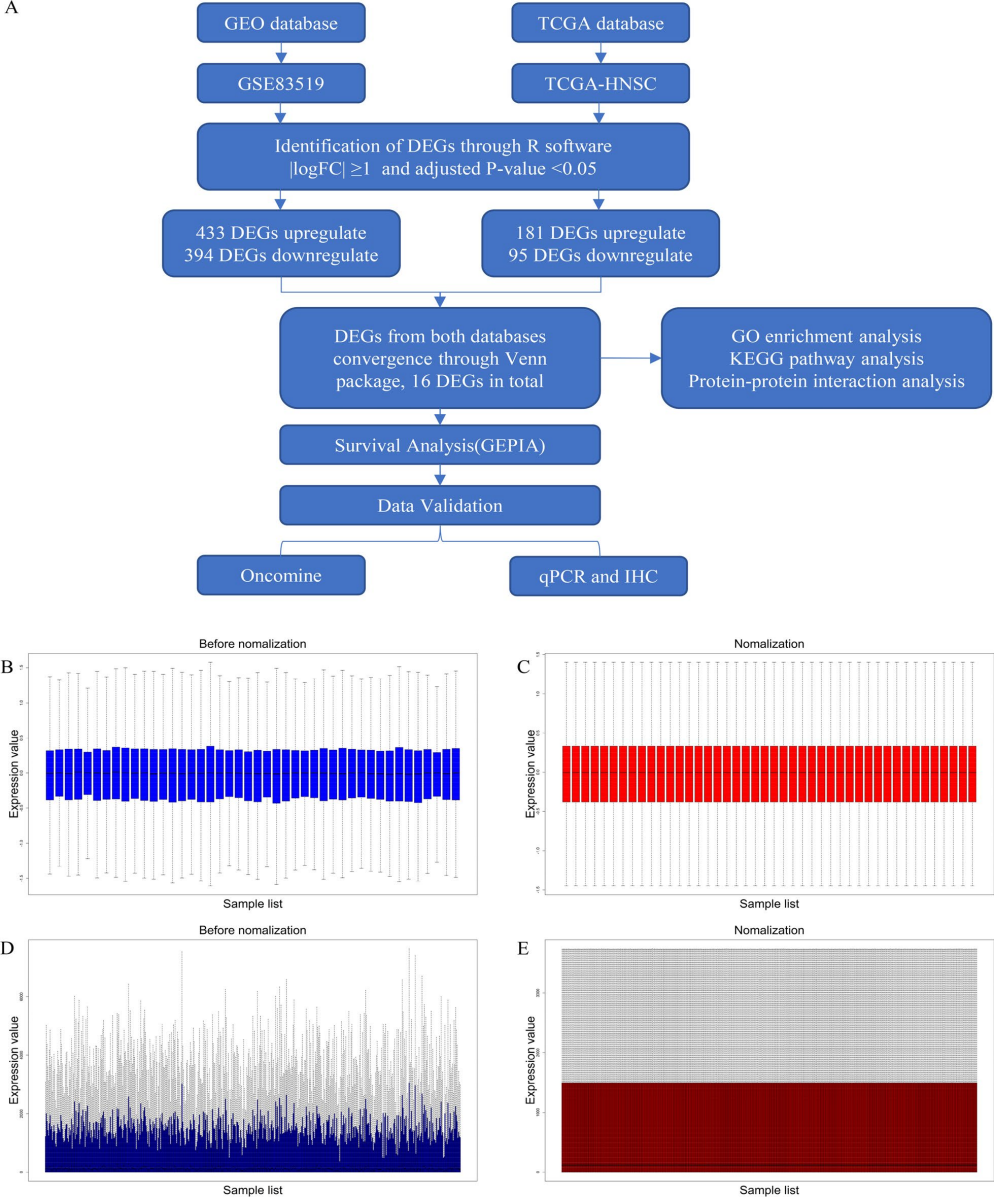
Flowchart and microarray data of the study. **A** Flowchart showing bioinformatics analysis of GEO and TCGA. **B**–**E** Standardization of gene expression. **B** Raw expression data from GSE83519. **C** Normalized expression data from GSE83519. **D** Raw expression data from the TCGA-HNSC dataset. **E** Normalized expression data from the TCGA-HNSC dataset

**Fig. 2:**
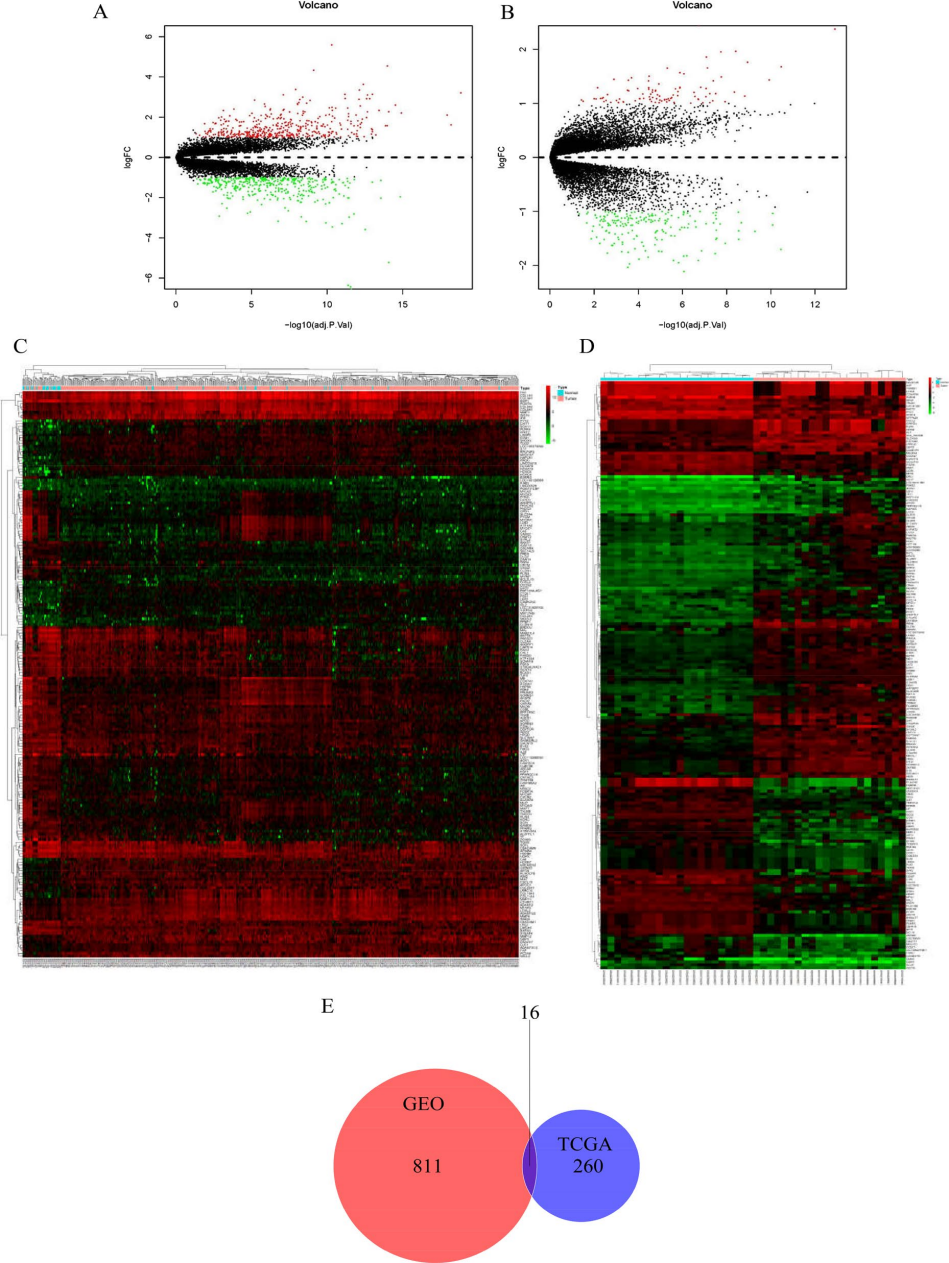
Identification of DEGs from two datasets. **A** Volcano map of 827 DEGs from GSE83519. **B** Volcano map of 276 DEGs from TCGA-HNSC. The red and green spots indicate up- and downregulated genes, respectively. The black spots represent the genes expressed with no significant difference between normal and tumour tissues. **C** Heatmaps of the top 200 | logFC | DEGs from GSE83519. **D** Heatmaps of the top 200 | logFC | DEGS from TCGA-HNSC. DEGs from the red and green plots indicate up- and downregulated genes, respectively. The black plots represent no significant difference in the expression between normal and tumour tissues. **E** Venn diagram showing the IDEGs from the two databases. Red area: genes found in the GEO dataset only; blue area: genes found in the TCGA dataset only; intersecting area: DEGs obtained from both databases

Both HNSCC expression datasets were standardized, and the results are shown in Fig. 1. According to the analysis by several R software packages, 827 DEGs were obtained from the GSE83519 dataset. A total of 433 upregulated genes and 394 downregulated genes were shown in the cluster analysis of these DEGs. When the TCGA-HNSC data were screened by the Limma package, 276 DEGs were extracted, which left 181 genes upregulated and 95 genes downregulated. The DEGs from both datasets are shown in the volcanic maps (Fig. 2A, B). Furthermore, DEGs from the two datasets were sorted by the absolute value of logFC. The top 200 genes of each dataset are shown in cluster heatmaps (Fig. 2C, D).

The Venn Diagram R software package was used for the selection of IDEGs from two databases, and a Venn map (Fig. 2E) was generated. Ultimately, 811 genes were found in GSE83519 only, and 260 genes were only obtained from TCGA-HNSC, whileand 16 IDEGs shown were obtained and are shown in Table 2.

**Table 2:** Differentially expressed genes from two datasets associated with HNSCC

Gene Symbol	LogFC	adj.P.Val
ATP6V0A4	3.63156	4.03E−13
TMPRSS11E	2.84745	6.25E−06
CYP2C18	2.69375	2.27E−09
CEACAM5	2.14075	1.11E−07
FMO2	1.9352	1.19E−06
CLCA4	1.92856	2.01E−07
ESM1	1.71976	4.15E−10
MYRIP	1.69976	1.42E−11
CEACAM6	1.34004	2.09E−04
MAOB	1.29486	8.33E−09
SPRR3	1.18998	1.05E−02
SYNPO2L	1.09951	2.71E−02
HOXC4	−1.2614	4.44E−04
ESRRG	−1.362	4.16E−08
MMP12	−2.2606	7.07E−11
ISG15	−2.8186	1.69E−12

Sixteen differentially expressed genes were screened out from both datasets and ranked by decreasing LogFC

*LogFC* Log fold change, *adj*.*P*. Val adjust P value

### GO enrichment analysis, KEGG signalling pathway analysis and PPI analysis of IDEGs

**Fig. 3:**
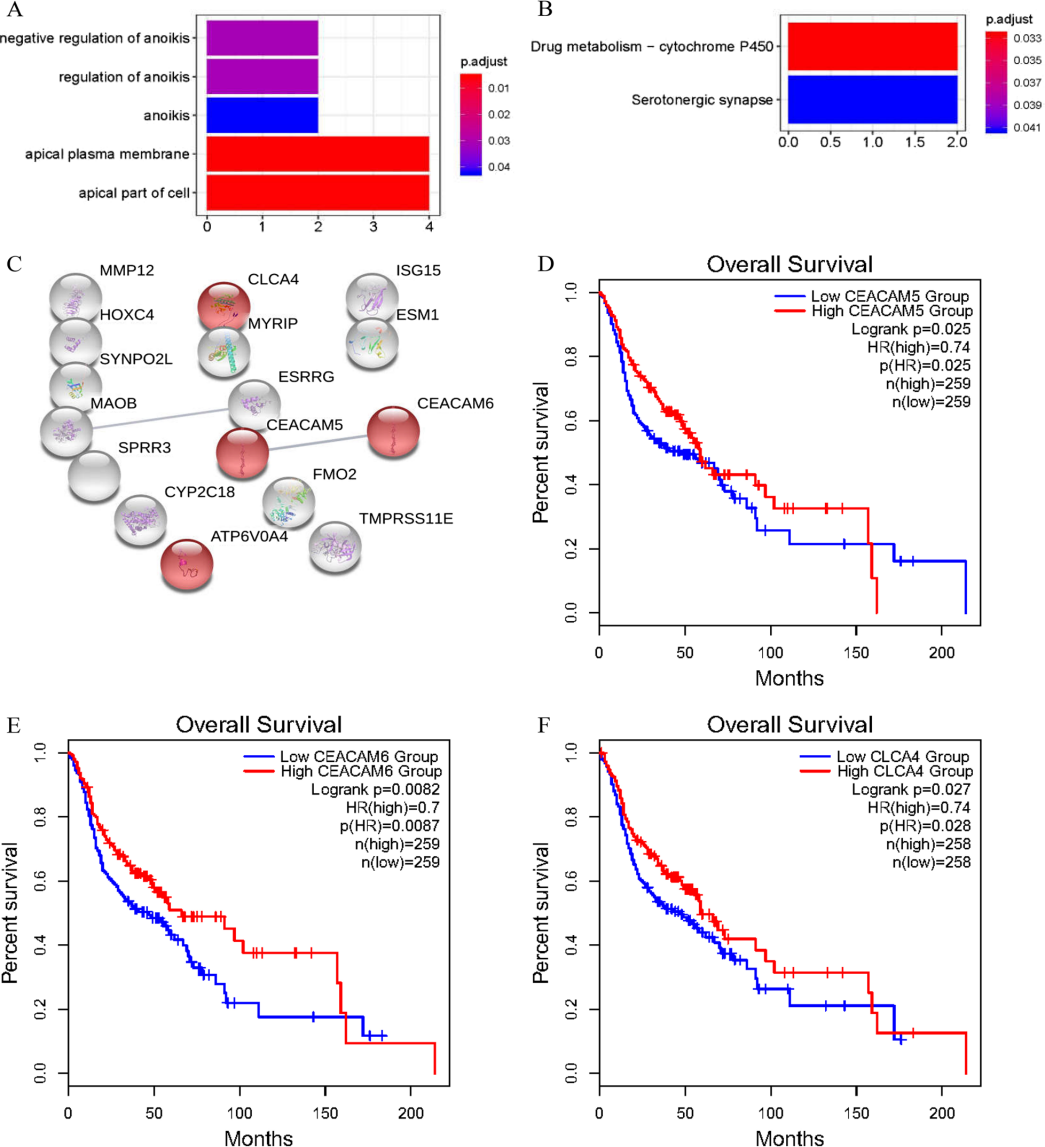
Further analysis of IDEGs to screen out key genes. **A** Significantly enriched GO terms of IDEGs. **B** Significantly enriched KEGG terms of IDEGs. **C** PPI network of IDEGs. Notes: Circles represent genes, lines represent the interaction between genes, and the shape within the circle represents protein structure. Red circles represent genes enriched in the GO term apical plasma membrane. **D** Survival analysis of CEACAM5 related to the overall survival rate of HNSCC patients (HR = 0.74, P = 0.025). **E** Survival analysis of CEACAM6 related to the overall survival rate of HNSCC patients (HR = 0.7, P = 0.00087). **F** Survival analysis of CLCA4 related to the overall survival rate of HNSCC patients (HR = 0.74, P = 0.028)

Generally, the GO enrichment analysis results contained three parts: biological process (BP), cell composition (CC) and molecular function (MF). GO terms were listed in ascending order according to the *q* values. Only the terms with both an adjusted *P*-value and *q* value < 0.05 were considered significantly enriched genes. In this study, the IDEGs were enriched in negative regulation of anoikis, regulation of anoikis and anoikis from BP, apical plasma membrane, and apical part of cell from CC. The results are shown in Table 3 and Fig. 3A.

**Table 3:** GO enrichment analysis and KEGG pathway analysis of differentially expressed genes associated with HNSCC

Terms	Description	adj.P.Val	q.Val	Count
GO terms				
GO:0043276	Anoikis	0.042	0.030	2
GO:2000209	Regulation of anoikis	0.031	0.022	2
GO:2000811	Negative regulation of anoikis	0.031	0.022	2
GO:0045177	Apical part of cell	0.0057	0.0041	4
GO:0016324	Apical plasma membrane	0.0055	0.0040	4
KEGG terms				
hsa04726	Serotonergic synapse	0.041	0.023	2
hsa00982	Drug metabolism-cytochrome P450	0.033	0.018	2

GO terms and KEGG pathway terms enriched with differentially expressed genes and ranked by decreasing adj.P.Val

*GO* Gene Ontology, *KEGG* Kyoto Encyclopedia of Genes and Genomes, *adj.P. Val* adjust P value, *q. Val* q value

The KEGG signalling pathway results were also sorted in ascending order according to the *q* value, and further screening was performed when the adjusted *P*-value and *q* value were simultaneously less than 0.05. The KEGG analysis showed that the IDEGs converged in two pathways, drug metabolism-cytochrome P450 and serotonergic synapse. All enriched pathways are reported in Table 3, and significantly enriched pathways are shown in Fig. 3B.

The IDEGs were then introduced into the online database String. Relevant PPIs were revealed and visualized, including 16 nodes and 2 edges in Fig. 3C.

### Survival analysis

When using GEPIA2 for survival analysis of the IDEGs, we calculated the correlation parameters between the overall survival (OS) rate of HNSCC patients and gene expression levels. The OS rates of patients with lower expression of CEACAM5 (*P* = 0.025), CEACAM6 (*P* = 0.0087) and CLCA4 (*P* = 0.028) were significantly lower than those of patients with higher expression, as shown in Fig. 3D–F. No significant correlation between HNSCC prognosis and the expression levels of the other 13 genes was found (*P* > 0.05). Therefore, CEACAM5, CEACAM6 and CLCA4 were identified as key genes.

### Verification of key genes

**Fig. 4:**
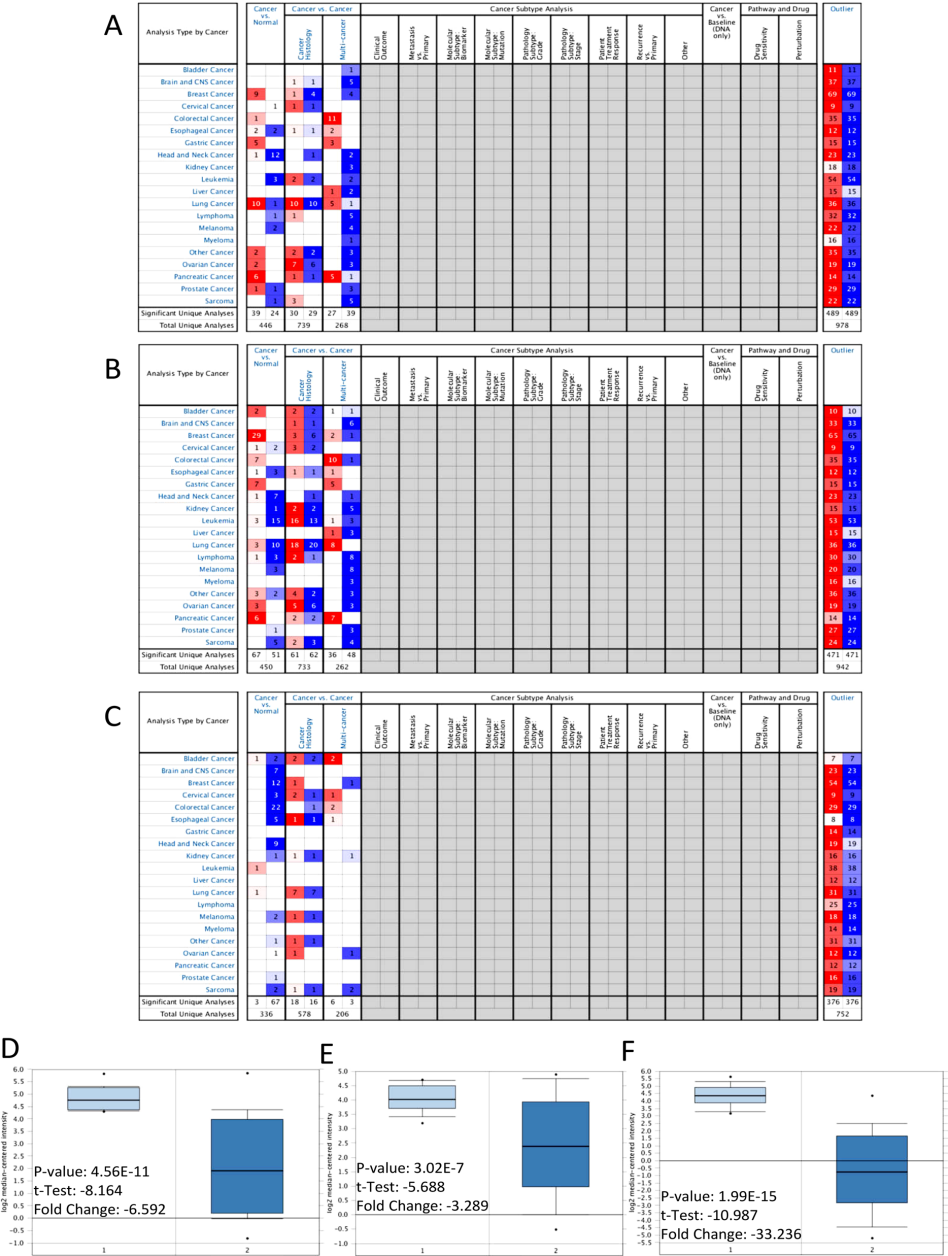
Expression levels of the three key genes in the Oncomine database. **A** CEACAM5 mRNA expression was significantly lower in HNSCC. **B** CEACAM6 mRNA expression was significantly lower in HNSCC. **C** CLCA4 mRNA expression was significantly lower in HNSCC (cell colour represents the best gene rank percentile). **D** CEACAM5 was significantly lower in HNSCC in Ginos Head-Neck. **E** CEACAM6 was significantly lower in HNSCC in Ginos Head-Neck. **F** CLCA4 was significantly lower in HNSCC in Ginos Head-Neck (1: Uvula; 2: HNSCC)

The reliability of key genes was verified by Oncomine. The expression levels of CEACAM5, CEACAM6 and CLCA4 in this database were downregulated in HNSCC specimens compared to adjacent noncancerous tissues (Figs. 4A–C, P < 0.05 and absolute value of fold change > 2). Red cells represent mRNA expression levels higher in HNSCC than in adjacent noncancerous tissues, while blue cells represent lower expression levels. The depth of colour is determined by the top gene percentile included in each cell. The darker colours represent higher percentiles. By reviewing the original expression data of Ginos Head-Neck, one of the HNSCC datasets in Oncomine, we found that all three key genes were significantly downregulated (P < 0.05 and absolute value of fold change > 2) in the Oncomine HNSCC datasets (Figs. 4D–F).

IHC and real-time PCR were performed to evaluate the protein and RNA expression levels of CEACAM5/6 and CLCA4 in five pairs of matched LC, HPC and OPC samples and adjacent noncancerous tissues.

**Fig. 5:**
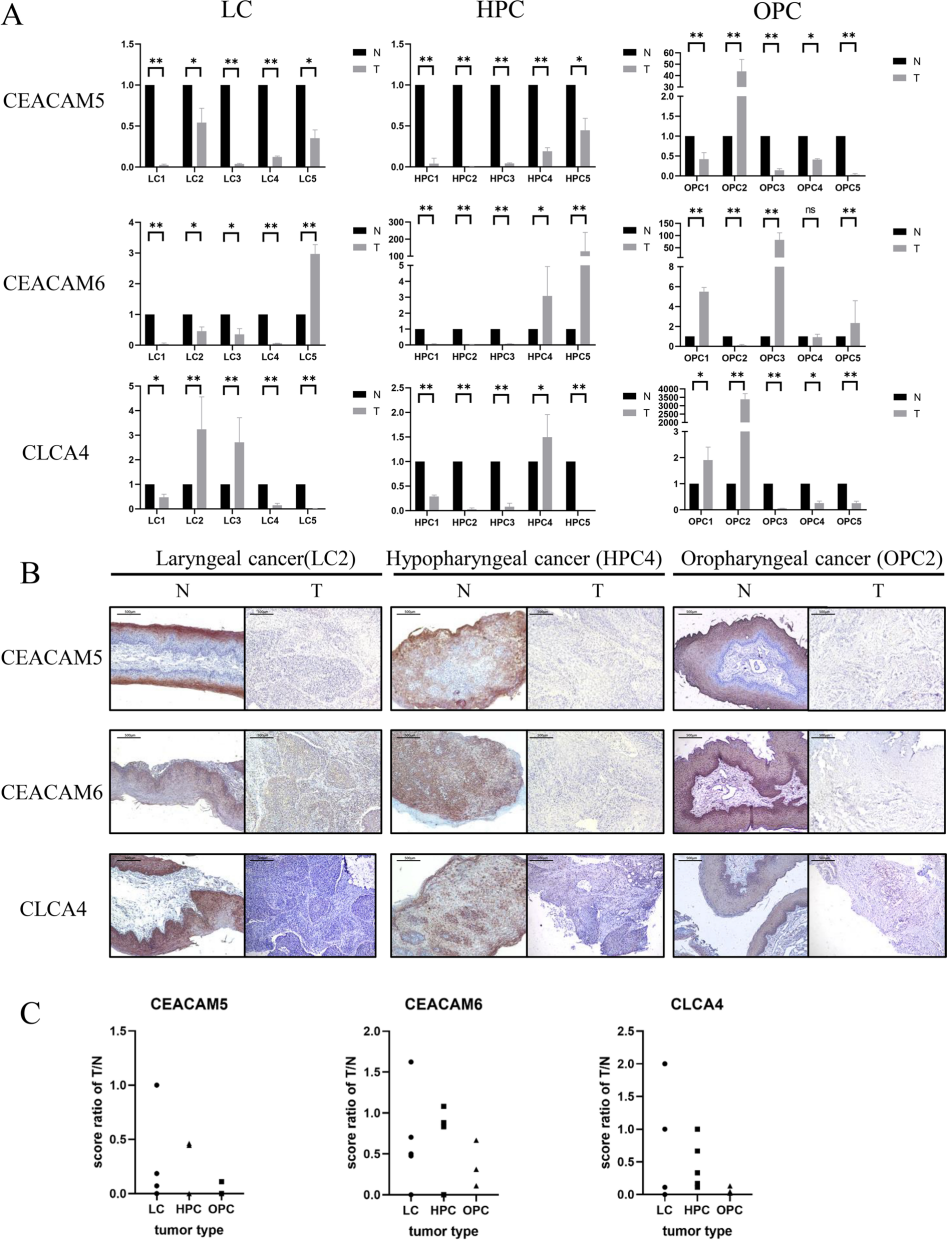
Expression levels of the three key genes in different HNSCC tissues. **A** Real-time PCR of CEACAM5, CEACAM6 and CLCA4 in laryngeal carcinoma, hypopharyngeal carcinoma and oropharyngeal carcinoma. The relative mRNA expression levels of these three genes in most HNSCC tissues were lower than those in adjacent noncancerous tissues (scale bars represent the mean ± SEM, Student’s t-test, *P < 0.05, **P < 0.01). **B** IHC of CEACAM5, CEACAM6 and CLCA4 in laryngeal carcinoma, hypopharyngeal carcinoma and oropharyngeal carcinoma. The protein expression levels of these three genes were lower than those in adjacent noncancerous tissues. **C** Semiquantification analysis of immunohistochemistry of each laryngeal carcinoma, hypopharyngeal carcinoma and oropharyngeal carcinoma tissue and paired adjacent normal tissue. Most of the tumour tissues in this study showed less staining than adjacent normal tissues. Abbreviations: LC: laryngeal carcinoma; HPC: hypopharyngeal carcinoma; OPC: oropharyngeal carcinoma

The real-time PCR results are shown in Fig. 5A. The relative mRNA expression levels of CEACAM5 in all LC, HPC and 4 of 5 OPC tissues were lower than those in adjacent noncancerous tissues. The relative mRNA expression levels of CEACAM6 in 4 of 5 LC, 3 of 5HPC and OPC tissues were downregulated compared to adjacent noncancerous tissues. CLCA4 was expressed at significantly lower levels in 4 of 5 HPC, 3 of LC and OPC tissues (P < 0.05). The protein expression levels of the three key genes were detected by IHC. Representative pairs of tissues from each type of carcinoma are shown in Fig. 5B. The semiquantification analysis of IHC results is shown in Fig. 5C. The score ratio represents the ratio of scores of paired HNSCC/adjacent normal tissues. A case with a score ratio below 1 indicates that there is less staining in tumour tissue than in adjacent normal tissue.

## Discussion

The GEO database, created by the National Biotechnology Information Center (NCBI) in 2000[16], is an open access database with tumour and nontumour gene expression data [26]. GEO also provides tools such as GEO2R (https://www.ncbi.nlm.nih.gov/geo/geo2r/) that allow users to perform complicated analyses and to visualize gene expression data relevant to their specific interest [15]. TCGA, a project of the National Institutes of Health (NIH), aims to use genome analysis technology to explore the genetic changes in cancer and to provide publicly available gene-level data to help improve diagnosis or treatment levels against cancer [27]. Compared with the GEO database, data from TCGA are more systematic and comprehensive, and many websites also provide TCGA data analysis functions [27]. In our study, the gene expression data of 589 samples from 522 HNSCC patients in total were downloaded from both databases for analysis.

For HNSCC, since the release of TCGA-HNSC in 2015, more than 1000 related articles have been published. Biomarkers [28], molecular landscape [29], miRNA signatures [30], pathways [31] and other genomic research studies have been reported. These studies are helpful to understand the molecular mechanism of the occurrence and development of HNSCC and are of great significance for future treatment.

In our study, bioinformatics technologies were used to discover HNSCC-related genes from two databases instead of focusing on a single genetic event or cohort study, as in most previous studies. Sixteen IDEGs in total were screened from these two datasets, and they mainly involved the GO biological function term regulation of anoikis, the GO cellular component term apical part of the cell, and the KEGG pathway terms drug metabolism–cytochrome P450 and serotonergic synapse. The survival analysis of IDEGs identified 3 key genes, CEACAM5, CEACAM6 and CLCA4, that significantly correlated with the overall survival of HNSCC. Among them, CEACAM5 and CEACAM6 are enriched in the regulation of anoikis. All three genes were enriched in the apical plasma membrane and apical part of the cell. The relationships between the three genes and digestive malignancies have been confirmed, but few studies of HNSCC have been reported, and their function and mechanism have not been fully elucidated.

CEACAM5, also called CEA (carcinoembryonic antigen), is a major marker of progression and metastasis in digestive malignancies such as colorectal and pancreatic cancers. CEACAM5 is the only CEA family member that is widely accepted as a tumour marker and tumour recurrence indicator, especially for colorectal cancer. CEACAM5 overexpression has also been reported in other malignant tumours, such as gastric cancer [32], breast cancer [33], and pancreatic cancer [34]. However, the effect of CEACAM5 in HNSCC is controversial. Sarina Cameron et al. reported that CEACAM5 overexpression increases tumour growth and tumorigenicity by inhibiting PI3K/AKT-dependent apoptosis of HNSCC [35]. However, other HNSCC genome sequencing results showed that the CEACAM5 expression level was significantly downregulated in HNSCC [36, 37]. In our study, IHC and real-time PCR were performed on 5 pairs of LC, HPC and OPC samples and their adjacent noncancerous tissues. CEACAM5 was significantly downregulated in most HNSCC tissues, suggesting that CEACAM5 overexpression may inhibit HNSCC occurrence and development.

CEACAM6 (CD66c or NCA-90) is a nonspecific cross-reactive glycoprotein antigen that has a common antigenic determinant with CEACAM5. CEACAM6 is highly expressed in many human solid tumours but varies with different tissue types [38]. Similar to CEACAM5, CEACAM6 overexpression was considered a potential driving force of pancreatic cancer progression [39]. Additional studies on digestive system cancers found that CEACAM6 could promote invasion and metastasis through epithelial-to-mesenchymal transition (EMT) by activating the PI3K/AKT signalling pathway [40]. CEACAM6 has been regarded as a potential biomarker or therapeutic target for different malignancies. However, the situation is different in LC. A recent study showed that the expression level of CEACAM6 in LC tissues was lower than that in adjacent noncancerous tissues [41]. The relationships between CEACAM6 and other types of HNSCC have yet to be reported. Here, we found that in addition to LC, CEACAM6 was also downregulated in HPCs and OPCs. A significant negative correlation between these two genes and the prognosis of HNSCC was also found.

CLCA4, a widely recognized tumour suppressor gene, is considered an inhibitor of invasion, migration and EMT in hepatocellular carcinoma [39] and colorectal cancer[42]. However, there is a lack of convincing research on the relationship between CLCA4 and HNSCC. In this study, the role of CLCA4 as a tumour inhibitor in the occurrence and development of HNSCC was verified. CLCA4 was significantly downregulated in HNSCC, and patients with low CLCA4 expression levels statistically significantly lived longer.

Interestingly, the expression levels of these three key genes in tumour and adjacent noncancerous tissues were not completely consistent at the mRNA and protein levels, which may be due to posttranscriptional modification and warrants future investigations. Furthermore, the expression levels of these three genes screened from databases have been identified to be downregulated in most cancer tissues at both the mRNA and protein levels, meaning they have the potential to become biomarkers for HNSCC. However, this study does have some limitations. The relationships between the three key genes and the clinical stage of HNSCC patients are still unclear. IHC and statistical analyses of a larger number of samples, together with molecular biological experiments, will be performed in the future. Additionally, because nasopharyngeal carcinoma (NPC) is different from other head and neck malignancies in terms of epidemiology, pathology, natural history, and treatments, NPC is beyond the scope of this study [43].

## Conclusion

In conclusion, key genes and important signalling pathways or molecules were identified by comprehensively analysing the gene expression data from GSE83519 and TCGA-HNSC, which could potentially be screened as new biomarkers for HNSCC. In the future, the functions of the key genes related to HNSCC will be explored and confirmed in molecular biological experiments.

## Data Availability

The datasets used and/or analysed during the current study are available from the corresponding author upon reasonable request.

## Abbreviations

HNSCC: Head and neck squamous cell carcinoma
RNA-seq: RNA sequencing
GEO: Gene Expression Omnibus
TCGA: The Cancer Genome Atlas
DEGs: Differentially expressed genes
GPL: Gene Platform
GSE: Gene Series
TCGA-HNSC: HNSCC project of The Cancer Genome Atlas
logFC: Log fold-change
IDEGs: Intersected differentially expressed genes
GO: Gene Ontology
BP: Biological process
CC: Cellular component
MF: Molecular function
KEGG: Kyoto Encyclopedia of Genes and Genomes
PPI: Protein–protein interaction
LC: Laryngeal carcinoma
HPC: Hypopharyngeal carcinoma
OPC: Oropharyngeal carcinoma
CEACAM5: Carcinoembryonic antigen-related cell adhesion molecule-5
CEACAM6: Carcinoembryonic antigen-related cell adhesion molecule-6
CLCA4: Chloride channel accessory 4
IHC: Immunohistochemistry
OS: Overall survival
NPC: Nasopharyngeal carcinoma
